# Evaluating persistence of shape information using a matching protocol

**DOI:** 10.3934/Neuroscience.2018.1.81

**Published:** 2018-02-03

**Authors:** Ernest Greene, Michael J. Hautus

**Affiliations:** 1 Department of Psychology, University of Southern California, Los Angeles, USA; 2 School of Psychology, University of Auckland, Auckland, New Zealand

**Keywords:** shape recognition, shape encoding, visual persistence, working memory

## Abstract

Many laboratories have studied persistence of shape information, the goal being to better understand how the visual system mediates recognition of objects. Most have asked for recognition of known shapes, *e.g*., letters of the alphabet, or recall from an array. Recognition of known shapes requires access to long-term memory, so it is not possible to know whether the experiment is assessing short-term encoding and working memory mechanisms, or has encountered limitations on retrieval from memory stores. Here we have used an inventory of unknown shapes, wherein a string of discrete dots forms the boundary of each shape. Each was displayed as a target only once to a given respondent, with recognition being tested using a matching task. Analysis based on signal detection theory was used to provide an unbiased estimate of the probability of correct decisions about whether comparison shapes matched target shapes. Four experiments were conducted, which found the following: a) Shapes were identified with a high probability of being correct with dot densities ranging from 20% to 4%. Performance dropped only about 10% across this density range. b) Shape identification levels remained very high with up to 500 milliseconds of target and comparison shape separation. c) With one-at-a-time display of target dots, varying the total time for a given display, the proportion of correct decisions dropped only about 10% even with a total display time of 500 milliseconds. d) With display of two complementary target subsets, also varying the total time of each display, there was a dramatic decline of proportion correct that reached chance levels by 500 milliseconds. The greater rate of decline for the two-pulse condition may be due to a mechanism that registers when the number of dots is sufficient to create a shape summary. Once a summary is produced, the temporal window that allows shape information to be added may be more limited.

## 1. Introduction

*Iconic memory has often been likened to a sensory store whose contents drain away rapidly as soon as the influencing stimulus is turned off*. Vincent Di Lollo [1]

It is well known that the human visual system can identify shapes using minimal cues. This can be demonstrated in various ways, including by displaying them out of focus [2],[3], reducing resolution in the image [4], or by showing only a portion of the boundary [5]–[7]. The present laboratory has examined encoding of shape information using sparse sampling of boundary dots, with respondents being asked to identify common objects [8],[9] as well as letters [10],[11]. These studies have provided useful insights into visual mechanisms. However, asking for recognition of known shapes leaves open the question of whether the treatment conditions are affecting early shape encoding mechanisms, or are acting on long-term memory retrieval.

One can reduce the ambiguity by using shapes that are “unknown,” meaning that they are relatively dissimilar to any known shape or object. One can test recognition by providing two successive shape displays, the first being designated as the target shape and the second being a comparison shape that either matches the target or is a non-matching shape. Reducing the density of the boundary markers, *i.e*., providing only a spaced pattern of dots, precludes a ceiling effect that would likely occur if all the boundary dots were shown. One further avoids a contribution from long-term memory if each unknown shape is shown only once as a target. The respondent decides whether the two shapes that are displayed on a given trial are the “same” or are “different.” After completion of the full experiment with a given set of treatment conditions, the data are submitted to quantitative methods that are commonly known as signal detection analysis (or decision theory). This analysis provides an unbiased measure of the validity of the decisions. If the decisions are significantly above chance, one can assert that the task conditions influenced the shape encoding process and/or working memory. Using shapes that are not stored in long-term memory provides a better method for assessing early shape processing mechanisms.

Prior findings using this unknown-shape protocol found non-chance recognition of the target shape when the comparison shapes displayed extremely sparse boundary markers [12]. The targets were displayed at 100% density and comparison shapes were varied in density. There was a progressive decline of recognition as the density was reduced, but it was still above chance when only 5% of the dots were displayed. The reported experiments further demonstrated translation, size and rotation invariance. Each target shape was seen only once and the judgment was rendered within a few moments after the comparison shape was flashed. This indicates that shapes are encoded very quickly by the nervous system, and the shape summary can be translated, rotated or resized, as needed, for identification of a given shape.

The Greene & Hautus [12] experiments displayed all the dots in the boundary of each target shape, providing 100% density, then assessed recognition using low-density comparison shapes. Here, Experiment 1 examined encoding and recognition where both the target shapes and comparison shapes were reduced in density. Experiment 2 varied the interval between the target and comparison shapes to determine whether there was decay of shape information. Experiments 3 and 4 used two different protocols that call for sequential display of target dots, providing information about the interval across which shape information can be integrated.

## 2. Methods

### 2.1. Authorization and respondent participation

The experimental protocols were approved by the USC Institutional Review Board. Respondents were recruited from the USC Psychology Subject Pool. Thirty-two respondents provided the matching judgments, eight in each of the four experiments. Each respondent was told that participation was completely voluntary, and that they could end the test session at any time without any form of recourse or penalty.

### 2.2. Display equipment

Shape boundary dots were displayed on a 64 × 64 array of light-emitting diodes (LEDs), designated as the “display board.” Respondents viewed the board from a distance of 3.5 m. At this distance, the visual angle of a given dot is 4.92 arc′, dot to dot spacing is 9.23 arc′, and the total span of the array (center-to-center) is 9.80 arc°. These LEDs emit at 630 nm, which appears as bright red. The dots forming the boundary of a given pattern can be displayed one at a time, in any order, or as simultaneous light emission from a specified subset of dots. For the present experiments, each dot to be displayed was flashed for 10 µs at an intensity of 1000 µW/sr. Ambient room illumination was 10 lx, which is mesopic, so the dots were quite salient.

Control of the display board is provided by a Mac G4 Cube, running custom Tk/tcl instructions. The program code is further interpreted as machine language by a PropoxMMnet101 microcontroller that runs at 16 MHz. This allows for 1µs temporal resolution of display timing.

### 2.3. Shape inventory and dot sampling

There were 480 unknown shapes in the inventory, each providing a string of adjacent dots at the boundary of the shape. Across the inventory, the number of dots used to form this boundary ranged from 100 to 269, with the mean number of dots being 166. At the 3.5 m viewing distance, shape spans range from 2.0 to 3.5 arc°, with mean distance being 2.6 arc°.

Each experiment displayed low-density versions of a given unknown shape. Which dots to include was based on a sampling protocol. First an address was chosen at random from the list, then successive addresses were included or not using an algorithm that maximized the net distance among dots. In other words, the protocol yielded a display wherein the dots were evenly spaced around the boundary at the density that had been specified.

### 2.4. Task protocols

**Figure 1.**
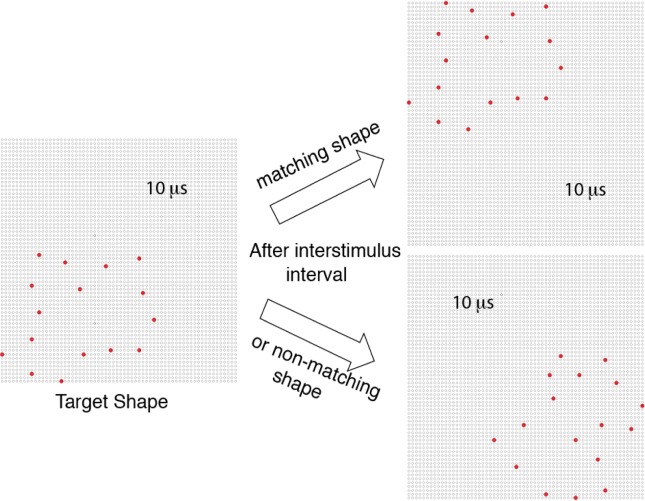
The experimental protocol displayed unknown shapes as a pattern of discrete dots that marked the outer boundaries of the shapes. The first experiment varied the amount of spacing of boundary markers, *i.e.*, the density, and the other three experiments used a 12% density as shown in this illustration. Shapes were sampled at random from the inventory, each being displayed as a target by flashing each dot in the pattern for 10 µs. This was followed by display of a randomly selected comparison shape that had either the same pattern of boundary markers (matching) or provided markers from a different shape (non-matching). Each comparison shape was also displayed for 10 µs. On a given trial, the target shape was displayed in one randomly selected corner of the display board and the comparison shape was shown in a different corner, also selected at random.

Each experiment randomly sampled the inventory to provide low-density “target shapes” that were always displayed first on a given trial. This was followed by a randomly selected “matching shape” that had the same pattern of boundary dots as the target shape, or was a low-density version of a different shape from the inventory, designated as a “non-matching shape” (see Figure 1). Matching and non-matching shapes are collectively described as “comparison shapes.” The density specified for a given trial was used for both the target shape and the comparison shape that was displayed. A given target shape or non-matching shape was displayed only once to a given respondent.

Across experiments, each display trial was preceded by a fixation target, consisting of the four dots in the center of the LED array providing steady emission of light at 0.2 µW/sr. The fixation dots were extinguished 200 ms prior to display of the target shape.

On a given trial, the target shape was positioned in a randomly selected corner of the display board, such that at least one boundary dot was in the outer row and column of the LED array. The comparison shape was then displayed in one of the other corners of the board, the location being chosen at random.

Experiment 1 used 320 target shapes and 160 non-matching comparison shapes. This experiment displayed low-density versions of the shapes across five percentage levels, these being 4, 8, 12, 16 and 20%. All the boundary dots of a given target or comparison shape were displayed simultaneously, and the comparison shape followed the target display at an interstimulus interval of 300 ms. The total number of display trials was 320, half showing matching shapes and half showing non-matching shapes, which provided 32 replications at each density level.

Experiment 2 again used 320 target shapes and 160 non-matching comparison shapes. All shapes were displayed at 12% density. Here the interstimulus interval was varied from 150 ms to 750 ms in 150 ms increments to determine whether the target shape information being held in working memory would decline. Here again, there were 32 replications at each of the five treatment levels.

Experiment 3 used 300 target shapes and 150 non-matching comparison shapes, reducing the total number of trials to assure that the task conditions would not exceed the time allowed for the test period. All shapes were shown at 12% density. For this experiment one of the six treatment conditions provided for simultaneous display of all the dots in the target shape. The other five levels displayed the dots one at a time, adjusting the interval between successive dots to meet a specified total time for showing them all. To be specific, total display times were 0, 100, 200, 300, 400 and 500 ms. The comparison shape was displayed at an interstimulus interval of 750 ms. For display of 300 target shapes and six levels of total display time, there were 25 replications at each treatment level.

Experiment 4 used 300 target shapes and 150 non-matching comparison shapes, displaying all shapes at 12% density. Here the dots in each low-density target shape were divided into two subsets using the following method. A dot was picked at random, and moving counterclockwise, successive dots were numbered. The odd numbered dots were assigned to one subset and the even numbered dots were assigned to the other. The dots in each subset were displayed simultaneously with a target interstimulus interval that varied from zero to 500 ms in 100 ms increments.

### 2.5. Task administration and response

Respondents were instructed to maintain their gaze on the fixation point prior to initiation of a given trial and to not move from that fixation as shape stimuli were being displayed. They were informed that the displays would briefly flash a pattern of dots that marked locations on the outer boundary of unknown shapes and that these shapes were unlikely to resemble recognizable objects. They were made aware that the dots would be spaced, but nonetheless would mark locations on the boundary of each shape. Respondents were also told that on each trial there would be two displays and the goal was to say whether the second shape was the same or different from the first. Respondents in Experiments 3 and 4 were further informed that the target dots could be shown as a sequence across an interval that could last up to half a second, and that the comparison shape would come after an interval that was even longer.

The experimenter initiated a given trial by clicking an on-screen button, which displayed the target/comparison stimulus set. The task was not speeded, but respondents generally offered a judgment within a second or two, saying either “Same” or “Different” as an indication of whether they thought the comparison shape matched the target shape. The experimenter recorded this answer by clicking the appropriate on-screen button, which logged the stimulus treatments that were used for that trial along with the judgment of the respondent. The experimenter was not provided any information about which treatment condition was used for a given display, nor did the respondent receive any feedback about whether the judgment was correct. In each of the experiments, all the trials for a given respondent were typically completed within 45 minutes.

### 2.6. Data analysis

Each respondent was able to complete the experiment for which he or she had volunteered, and no data was discarded. Evaluation of the data was based on signal detection theory, as described in detail in an earlier report [12]. As overview, signal detection theory provides a measure of performance, *d′*, that is corrected both for response bias and the structure of the task. The most commonly adopted formula, *d′* = *z*(*H*) − *z*(*F*), is appropriate for the standard detection-theory task, also called the yes-no task [13]. This formula is not suitable for our match-judgement task, which is formally a yes-no reminder task. We therefore based our calculations on the formula appropriate for this task, *d′* = √2(*z*(*H*) − *z*(*F*)), where *H* was the proportion of “same” judgments to matching shapes, *F* was the proportion of “same” judgments to non-matching shapes, and *z* (•) was the inverse-normal transform [14]. Values of *F* and *H* were adjusted (which would otherwise lead to *d′* = ± ∞ for values of *F* or *H* of either 0 or 1) using the log-linear correction [15] prior to calculation of *d′*.

The variance of each *d′* value was determined using similar calculations as in the tabulation method given by Miller [16] for the yes-no task. This was accomplished using customized software [17]. The calculated variance of *d′* was employed to generate confidence intervals, *e.g.*, *d′* ± 1.96 √σ^2^, where σ^2^ is the estimated variance.

When assessing performance across respondents, the data from different respondents should not be combined [18],[19]. Instead, the estimates of *d′* for each individual respondent was averaged to provide the group estimate. The variance of group *d′* was the sum of the *N* individual respondent variances divided by *N^2^*. The group variance allowed confidence intervals to be established around group *d′*.

Rather than presenting *d′* with confidence intervals, we present a more familiar proportion measure called *p*(*c*)*_max_*. This measure, like *d′*, corrects for response bias, with 0.5 reflecting performance and chance level and with judgments that are always correct being scored at 1.0. For our matching task, *p*(*c*)_max_ = (*d*′/(2√2)), where φ (•) was the cumulative normal probability distribution function. This formula facilitated transformation of estimates of *d′* and their confidence intervals to estimates of *p*(*c*)*_max_* and their confidence intervals, which we report and illustrate in our figures. Finally, to assess systematic changes in *p*(*c*)_max_ across conditions, linear contrasts consequent on repeated measures analysis of variance are reported.

## 3. Results

**Figure 2.**
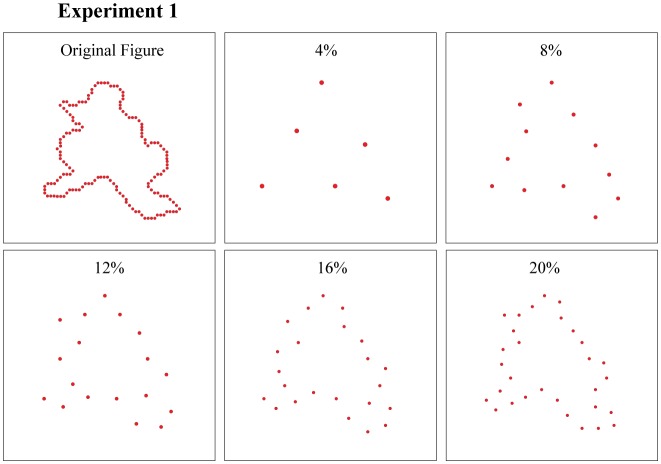
The upper left panel shows an example from the shape inventory with a full complement of dots marking the boundary. Experiment 1 chose sparse samples at five levels of density, as illustrated in the other panels, for display as target and comparison shapes, the same density being used for both on a given trial. Dot sizes have been adjusted in this illustration to compensate for the decline in perceptual salience as density is reduced. Flashed dots with mesopic levels of ambient lighting were readily perceived across all densities.

Experiment 1 varied the density (sparseness) of boundary dots in each display from 4% to 20%, with the target and comparison shapes being shown at the same density on a given trial (see Figure 2).

**Figure 3.**
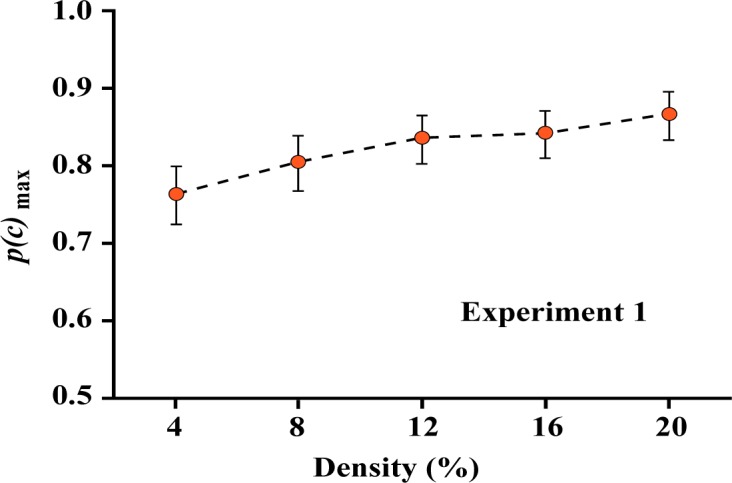
The probability of a correct matching judgment dropped as the density of boundary dots was reduced from 20% to 4%, but that probability was still at a very high level even at the lowest density. This differs from prior work [12] that found a steeper decline where the target was displayed at 100% density. The difference here was that low-density shape cues were provided for the target as well as comparison displays. Whereas a low-density pattern provides a less effective match to the full shape, it can be readily matched when the target and comparison shapes have corresponding densities.

Mean *p*(*c*)_max_ values, each being the average across the eight subjects, are plotted in Figure 3. One can see that the probability of making correct decisions was quite high and declined no more than 10% as a function of density. Clearly, the shape information provided by a sparse pattern of dots was quite sufficient to allow judgments with a high-probability of being correct.

Degree of overlap in the confidence intervals can be used to assess the significance of differences in *p*(*c*)_max_. While means systematically increase, the linear contrast was not significant (*F* (1,7) = 2.586, *p* = 0.152).

**Figure 4.**
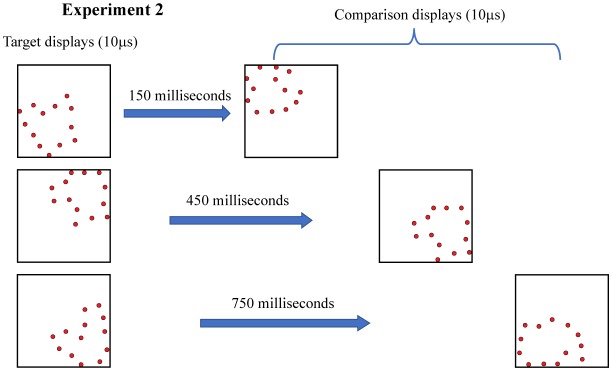
Experiment 2 used 12% density for all shape displays, fewer dots being shown here for illustration purposes. The interstimulus interval was varied across a range from 150 to 750 milliseconds, the goal being to determine the duration across which target information would persist. Three levels are illustrated here.

**Figure 5.**
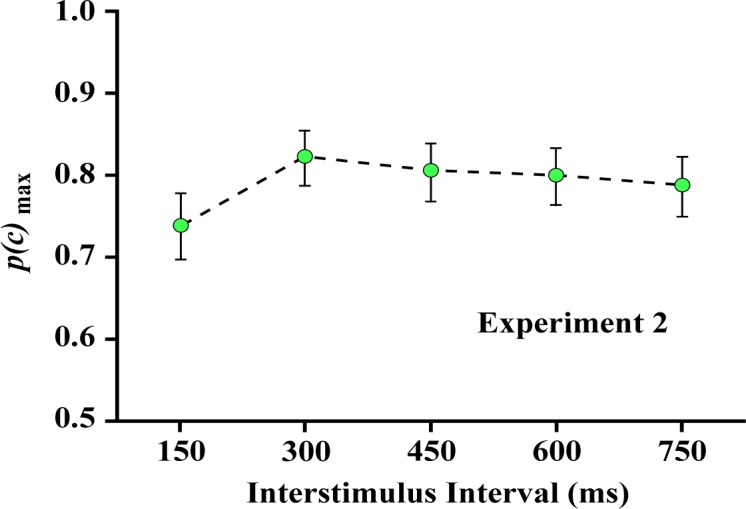
The probability of correct decisions was reduced somewhat at the shortest interstimulus interval, but otherwise the duration of that interval had very little effect on the probability of correct matching decisions.

Experiment 2 examined whether effectiveness of judgments would decline as a function of length of the interstimulus interval, as illustrated in Figure 4. As can be seen in Figure 5, judgments done with the interstimulus interval at 150 ms was significantly depressed, but there were high levels of correct judgment at other intervals out to 750 ms.

Here, the linear contrast was not significant (*F* (1,7) = 1.959, *p* = 0.204), but the quadratic contrast did reach significance (*F* (1,7) = 7.103, *p* = 0.032), giving further support to the *p*(*c*)_max_ differential at 150 ms. Additional analysis done with this treatment level removed did not find the linear component to be significant (*F* (1,7) = 2.630, *p* = 0.149).

**Figure 6.**
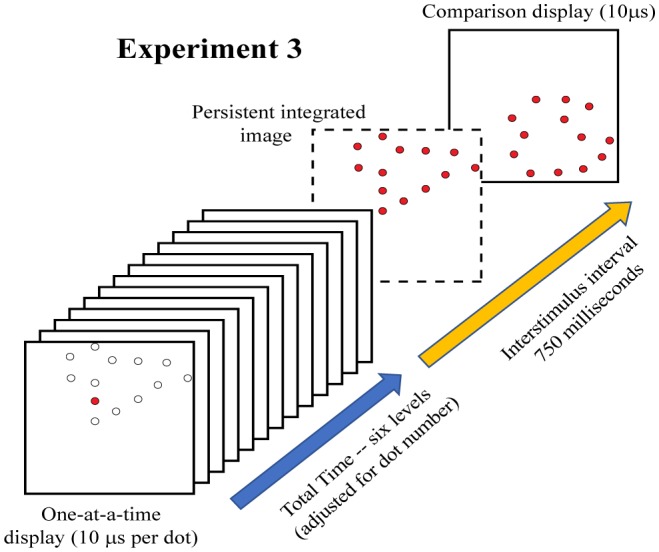
Experiment 3 successively displayed one dot at a time until all dots in the 12% sample were delivered. The first frame of the illustration shows one dot being flashed and the locations where dots will subsequently be flashed. The total time required to present all the sample dots was varied across six levels, from 0 to 500 milliseconds, to assess the time across which the stimulus information would be available in working memory. The dashed frame shows the complete pattern of dots that would be perceived if all dots in the target sequence persisted in working memory.

**Figure 7.**
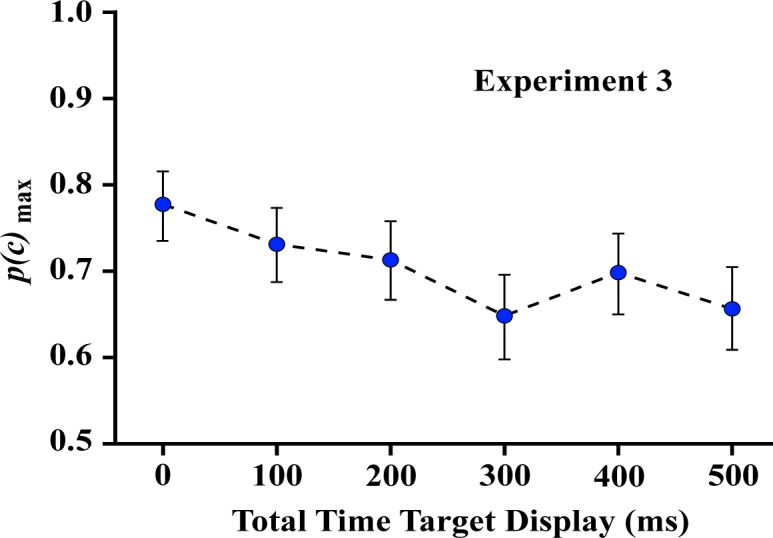
The probability of correct matching decisions declined as the total time for display of target dots was increased. Nonetheless, matching decisions were well above chance even when a 500-millisecond interval was used to display the full sequence of target dots. This shows that working memory maintains the information and provides for integration of the display sequence with only modest impairment of judgment across that interval.

Experiment 3 displayed each dot of a given target shape one at a time, requiring that all the dots be presented within a total interval of 0–500 ms, as illustrated in Figure 6. One can see from Figure 7 that this yielded a small drop in correct judgment probability across the range of total display times. These results indicate that the shape information is immediately available to inform judgment, and persists for 500 milliseconds with only modest decline in the probability of correct decisions.

The linear contrast for Experiment 3 was significant (*F* (1,7) = 18.34, *p* = 0.004). Nonetheless, one should note that *p*(*c*)_max_ declined little more than 10%, and the probability of a correct decision was well above chance even when the total time for display of all dots was 500 ms.

**Figure 8.**
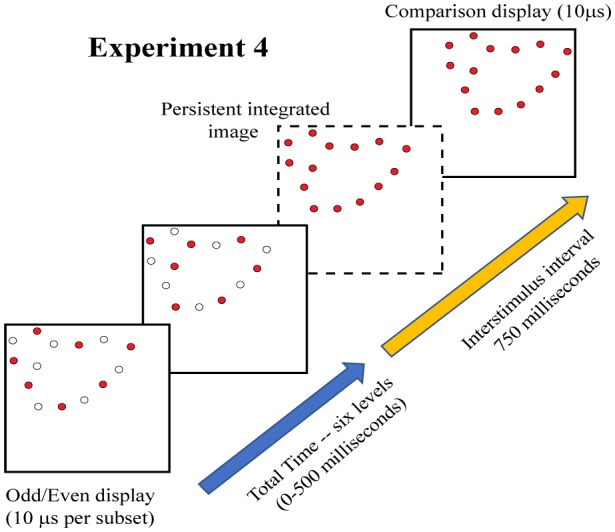
Experiment 4 displayed the 12% dot patterns in two successive displays. After numbering the dots in the pattern, those with odd numbers were displayed initially, followed by the dots that were specified with even numbers. Here again, the dashed frame shows the perceptual state that would be produced if the first pattern persists and is combined with the second pattern.

Experiment 4 was designed to re-evaluate that issue using a two-pulse protocol. As illustrated in Figure 8, low-density samples of target dots were divided into odd- and even-numbered subsets for display with various levels of temporal separation. The use of a matching task eliminates the need for a single-pulse control condition, given that total decay of information persistence is indicated when performance reaches chance levels.

**Figure 9.**
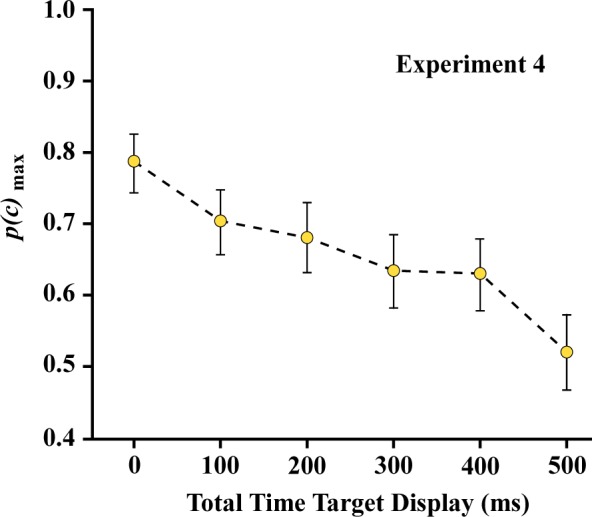
The decline in correct matching judgments was far steeper with the display conditions of Experiment 4. By 500 milliseconds, the *p*(*c*)_max_ rate was not significantly above chance. This suggests that a portion of the integration needed to provide accurate judgments is not based on persistence of stimulus information, *per se*, but also on the requirement to combine what is shown at one moment with what is displayed subsequently. It is hypothesized that the perceptual system registers the first target subset as a distinct package of information, and then closes off or restricts integration with the second subset that is displayed a few moments later.

One can see in Figure 9 that recognition of the target shapes declined substantially with increasing separation of the two subsets, reaching a level that was not significantly different from chance at 500 ms.

For Experiment 4 the linear contrast is significant (*F* (1,7) = 19.34, *p* = 0.003) supporting the linear decline apparent in Figure 9. This differs from what was found in Experiment 3, suggesting that something more than persistence of stimulus information is at work. Perhaps there is a cap on the ability to integrate separate packets of stimulus information that depends on more than the temporal separation. We will give further consideration to that matter after discussing other work that is relevant.

## 4. Discussion

The results of Experiment 1 differ substantially from earlier work [12] where all the dots in the target shape were displayed, *i.e.*, 100% density, and densities of comparison shapes were varied from 25% to 5%. In that experiment the ability to provide a correct matching judgment declined substantially as density was reduced. From that result one might reasonably infer that each boundary dot provides a small increment of shape information, and the display of more dots simply raises the amount of information being delivered.

The Experiment 1 results suggest a different concept. Here the target and comparison shapes were both displayed at the same density and judgments showed no significant impairment when both were displayed with a low density. The probability of correct judgments was essentially the same for 4% displays as with displays at higher-density. These results confirm an earlier report from this laboratory [20], wherein match judgments were comparable for 4% and 32% targets. It also provides a more complete assessment of the treatment effects by probing the range between 20% and 4%. These results argue against the concept that each dot has some limited ability to deliver shape information, and argues for a comparison process that is seeking to match the shape summary that was generated by the target.

Experiment 2 found very little reduction in match judgments with an interstimulus interval spanning 750 milliseconds, this being the longest interval that was evaluated. Finding consistently high judgment probabilities across a range of at least 750 milliseconds appears to be at odds with what was found by this laboratory for letter-recognition [10],[11]. The earlier experiments with letters used a two-pulse protocol wherein two complementary sparse (low density) dot subsets were successively displayed. The density of each subset was set very low, such that they would be minimally effective at eliciting recognition when a single subset was displayed alone. However, when the two were displayed in close succession, the information or partial memory activation produced by the first subset could be supplemented by the second, allowing a much higher level of recognition. The proportion of correct judgments declined as the interstimulus interval was increased, reaching the one-pulse level of recognition after 300–600 milliseconds (depending on specific task conditions).

These earlier findings attributed the decline in recognition to decay of shape information being held in working memory. The results from Experiment 2 call for a re-analysis of that conclusion. Here there was not much decay of shape information, *i.e.*, decline in probability of correct judgment, as the interstimulus interval was increased. It seems clear that working memory can retain the shape information for at least 750 milliseconds. The declines found for letter recognition may be specific to tasks requiring synthesis of complementary information, *e.g.*, a two-pulse protocol. Or the temporal window for retrieving information from long-term memory may be shorter. These alternatives will be given additional consideration below.

Experiment 3 displayed each dot of a given target shape one at a time, requiring that all the dots be provided within an interval that ranged from 0 to 500 milliseconds. Here the ability to correctly judge whether the comparison shape was the same or different depended on persistence of the information provided by each dot but also the ability of the visual system to integrate that information to yield a shape summary. It is possible that the duration of persistence for an individual dot would be shorter than for a full dot pattern. More likely, any decline that was manifested for intervals shorter than 750 milliseconds would be due to temporal restrictions on the integration process, *per se*. The judgments in Experiment 3 were well above chance across the full 500 millisecond interval, but there was a significant decline in *p*(*c*)_max_. Further, the probability of being correct at 500 milliseconds was significantly below what was found in Experiment 2 for that interval.

Greene & Ogden [21] used the one-at-a-time protocol to study recognition of known shapes, *e.g.*, animals, vehicles, furniture, tools, with the total time for display of all the dots varying between zero and 700 milliseconds. There was a linear decline in recognition, with the rate of decline depending on which shape was being judged. On average, shape recognition dropped by 50% across a total display time of 700 milliseconds. In Experiment 3 the recognition of unknown shapes declined by only 10% with a total display interval of 500 milliseconds. Finding that identification of unknown shapes declines much less than for known shapes suggests that the need to access long-term memory provides a substantial restriction on performance.

Experiment 4 used a two-pulse protocol, wherein target dots were divided into two complementary subsets and these were displayed with six levels of temporal separation. Here the performance declined at a much steeper rate than in Experiment 3, and reached the chance level of 0.5 with subset separations of 500 milliseconds. The differential is pertinent to the earlier question of whether the persistence of information for individual dots might be less than for dot patterns. If that were the case, one would expect the decline in performance for the one-at-a-time protocol to be steeper than for a two-pulse protocol. Finding the reverse now puts the focus on the integration process, and raises the question of why the temporal window would be more restrictive for integrating two complementary patterns.

Phillips [22] provided a seminal study of this process using randomly selected dot patterns wherein the size of the matrix (array) was varied. Two successive displays presented matching or non-matching patterns and respondents were required to judge whether the two were the same or different. Performance was fast and accurate if there was no shift in the location of dots and the interval between successive displays was 100 milliseconds or less. For those judgments, the visual system was likely drawing upon what Coltheart [23] described as “visible persistence,” which generally has been found to last for roughly 100 milliseconds. The impulse response of photoreceptors lasts for up to 100 milliseconds, and two near-threshold flashes can sum to allow high-probability recognition of letters across this same interval [10]. This is consistent with the proposition that retina provides the mechanism for visible persistence of stimulus information.

With an interstimulus interval beyond 100 milliseconds, Phillips [22] found that performance was slower, less accurate, and highly dependent on stimulus complexity. He did find persistence of stimulus information for at least 600 milliseconds with substantial declines at longer intervals. But even at 600 milliseconds there were significant differentials in judgment accuracy for 5 × 5 matrices compared to 8 × 8 matrices. The results from Experiment 1 found no meaningful difference in judgment probability as a function of dot density, which of course greatly alters the number of dots being presented. It is possible that the difference in result pivots on Phillips' use of random dots, whereas the present manipulation was of boundary dots. A decrease in density of boundary dots modifies the summary that is generated, but does not impair the system's ability to produce a summary.

Several reports from this laboratory used the two-pulse protocol to evaluate recognition of known shapes. Greene [24],[25] asked for recognition of namable objects using complementary low-density subsets and providing differentials of room illumination. The percentage of shapes that could be identified dropped from about 80% to roughly 60% over a 270-millisecond interval when the room was dark, but dropped into the 40% range within 90 milliseconds when the room was well lighted [24]. Subsequent work that added a dim condition found the declines to be extremely linear across the two-pulse interval. Recognition was in the 70% range at 0 milliseconds of pulse separation for all three levels of ambient illumination. It dropped to about 35% at 40 milliseconds for the bright room, 40% at 80 milliseconds for the dim room, and about 50% at 160 milliseconds for the dark room [25].

Subsequent work with recognition of letters used low-density subsets in a two-pulse protocol and dim room illumination [10],[11]. For these shapes the information persistence was found to be substantially longer, ranging from 300 to 600 milliseconds for some task conditions and potentially staying above the one-pulse range of hit rates for an interval that was even longer. Letters are heavily overlearned, so this might be a factor for the interval that allows for information integration. Also, there are only 26 letters in the inventory to be identified, whereas the number of namable objects is very large and open-ended.

Any conceptual model of image analysis and shape recognition can be partitioned into a plethora of functional steps, but at this point it appears that there are four major operations that can be distinguished. The first is encoding, wherein the elemental shape cues are converted into a shape summary. Some conditions allow this to be completed within tens of milliseconds, or no more than a hundred milliseconds, as discussed in articles cited previously [8]–[11]. The second mechanism provides a means hold stimulus information for an interval sufficient to achieve encoding, this being described as persistence. The persistence of shape cues can extend for the better part of a second, as evidenced by the results of Experiment 2. The third is a means to determine whether the summary process should be terminated or extended. The differentials found in Experiment 3 versus Experiment 4 suggest that this interval is somewhat determined by the number of cues, *e.g.*, boundary markers, that are provided at a given moment. A one-at-a-time display sequence is open-ended, which apparently does not elicit closure of the encoding process. Alternatively, the very low-density subsets of Experiment 4 provided dot patterns that could be “packaged” as a shape summary, which then greatly restricted the interval wherein information could be added. A fourth mechanism would be the process by which a shape summary can access memories, thus allowing for identification of known shapes. Based on prior work, this final step also appears to place restrictions on the integration interval, but at least that constraint is not relevant for the present work wherein the shapes were displayed only once and therefore do not require retrieval from long-term memory.

## 5. Conclusions

The present results demonstrate that manipulation of density, *i.e*., sparseness of boundary markers, does not produce significant differentials in recognition of unknown shapes if both the target shape and the comparison shape have the same level of density. The shape information is encoded and stored in working memory with no significant decline of that information for at least 750 milliseconds. Boundary location cues of a target shape can be delivered sequentially, *e.g.*, by displaying sparse dots one at a time, and shape recognition remains high for up to 500 milliseconds of total display time. However, if the same sparse pattern is divided into two subsets, with the dots in each being displayed simultaneously, there is a substantial drop in recognition across this same interval. This suggests that decay of information persistence is not the only reason that recognition would decline. There may be an active process of exclusion that protects the information that was gathered at one moment against disruption at a later moment. Apparently, this can occur even if the two sources of shape information are compatible.
